# Methanol poisoning during the COVID‐19 pandemic in Iran: A retrospective cross‐sectional study of clinical, laboratory, and brain imaging characteristics and outcomes

**DOI:** 10.1002/hsr2.1752

**Published:** 2023-12-12

**Authors:** Saeid Esmaeilian, Arash Teimouri, Sedighe Hooshmandi, Mohammad Hossein Nikoo, Seyed Taghi Heydari, Elham Mohajeri, Sina Bazmi, Reza Tabrizi, Meisam Hoseinyazdi

**Affiliations:** ^1^ Health Policy Research Center, Institute of Health Shiraz University of Medical Sciences Shiraz Iran; ^2^ Medical Imaging Research Center Shiraz University of Medical Sciences Shiraz Iran; ^3^ Noncommunicable Diseases Research Centre Shiraz University of Medical Sciences Shiraz Iran; ^4^ Student Research Committee Fasa University of Medical Sciences Fasa Iran; ^5^ Noncommunicable Diseases Research Center Fasa University of Medical Sciences Fasa Iran; ^6^ Clinical Research Development Unit, Valiasr Hospital Fasa University of Medical Sciences Fasa Iran; ^7^ USERN office Fasa University of Medical Sciences Fasa Iran; ^8^ Russell H. Morgan Department of Radiology and Radiological Science Johns Hopkins Hospital Baltimore Maryland USA

**Keywords:** brain CT, COVID‐19 pandemic, methanol poisoning, prognosis

## Abstract

**Objective:**

To evaluate the frequency and significance of brain imaging findings in methanol poisoning patients and to propose a criterion for prioritizing brain imaging.

**Methods:**

We retrospectively reviewed the data of 306 patients (286 men and 34 women, mean age 32.10 ± 9.9 years) with confirmed methanol poisoning who were admitted to two hospitals in Iran during the COVID‐19 pandemic. We analyzed their demographic, clinical, laboratory, and brain imaging data.

**Results:**

The main brain computed tomography (CT) scan findings were hypodensity in the putamen (11.1%), cerebellar nuclei (8.2%), diffuse cerebral edema (7.5%), and intracranial hemorrhage (ICH; 1.6%). These findings were associated with blood pH, Glasgow Coma Scale (GCS), renal failure, bicarbonate, oxygen, carbon dioxide, potassium, and glucose levels (*p* < 0.05). Poor prognosis was related to blindness, opium addiction, chronic alcohol use, hyperglycemia, and abnormal CT scans (*p* < 0.001 for all). The most predictive brain imaging findings for poor prognosis were hypodensity in the cerebellar nuclei, diffuse cerebral edema, and ICH.

**Conclusion:**

Brain imaging can provide valuable information for the diagnosis and management of methanol poisoning patients. We suggest that patients with severe acidosis, low GCS, low pH, low oxygen saturation, and high glucose levels should undergo brain CT scan as a priority.

## 1 INTRODUCTION

The consumption of methanol is highly toxic and can lead to the production of formic acid and formaldehyde during metabolism. Ingesting methanol can result in various adverse health conditions and even death.^1^ The metabolites of methanol have a significant impact on the gastrointestinal tract and central nervous system, causing symptoms such as metabolic acidosis, nausea, vomiting, and visual impairment.^1^, ^2^ This combination of vision impairment, gastrointestinal symptoms, and metabolic acidosis within 6–24 h is sometimes referred to as a trio.

Methanol poisoning is a relatively uncommon form of poisoning in the medical field, usually occurring due to accidental exposure.^3^, ^4^ However, there has been an increase in the incidence of methanol poisoning outbreaks, particularly in some Islamic nations. This rise in cases is often attributed to the prohibition of alcohol drinking in Islamic regulations.^5^ An outbreak of methanol poisoning was linked to the consumption of illegally manufactured beverages.^5^, ^6^ Additionally, during the COVID‐19 pandemic, there was a widespread belief in Iran that consuming alcoholic beverages could provide protection against the virus. Iran has been experiencing a significant number of methanol poisoning outbreaks, even before the COVID‐19 pandemic, making it a prominent location for such incidents in recent years.^5^, ^6^, ^7^, ^8^, ^9^, ^10^ These outbreaks have posed major challenges for healthcare providers in terms of diagnosis and treatment.^2^, ^7^

The etiology of methanol‐induced neurotoxicity remains unknown; however, it presents with a wide range of symptoms including loss of consciousness, seizures, intracranial bleeding, and cerebral edema.^11^, ^12^ A previous study has shown that intracranial events, although rare, have a significant impact on patient prognosis.^12^ Earlier investigations have reported the occurrence of hemorrhagic and non‐hemorrhagic necrosis in the basal ganglia, white matter, and widespread brain edema. Some nonspecific abnormalities, such as sub‐cortical hypodensity or intraventricular hemorrhage (IVH), have also been observed in case reports.^3^, ^6^, ^12^, ^13^, ^14^, ^15^, ^16^ Unfortunately, these previous studies had limited sample sizes, resulting in a lack of methanol poisoning‐related imaging data. Additionally, the specific or nonspecific nature of each imaging finding and its correlation with patient prognosis and potential treatment contribution remains unknown.

Methanol poisoning outbreaks in Iran and similar cultural countries have been well‐documented, often coinciding with other disease outbreaks like COVID‐19. However, there is currently a lack of appropriate protocols for prioritizing patients in need of advanced care, such as urgent dialysis or admission to the intensive care unit (ICU). This study aims to determine the frequency and significance of different brain findings and their clinical manifestations, as well as their impact on patient prognosis. By providing a clearer understanding of methanol poisoning, this research aims to assist policymakers and clinicians in developing effective policies and protocols for future outbreaks.

## 2 MATERIALS AND METHODS

**Figure 1:**
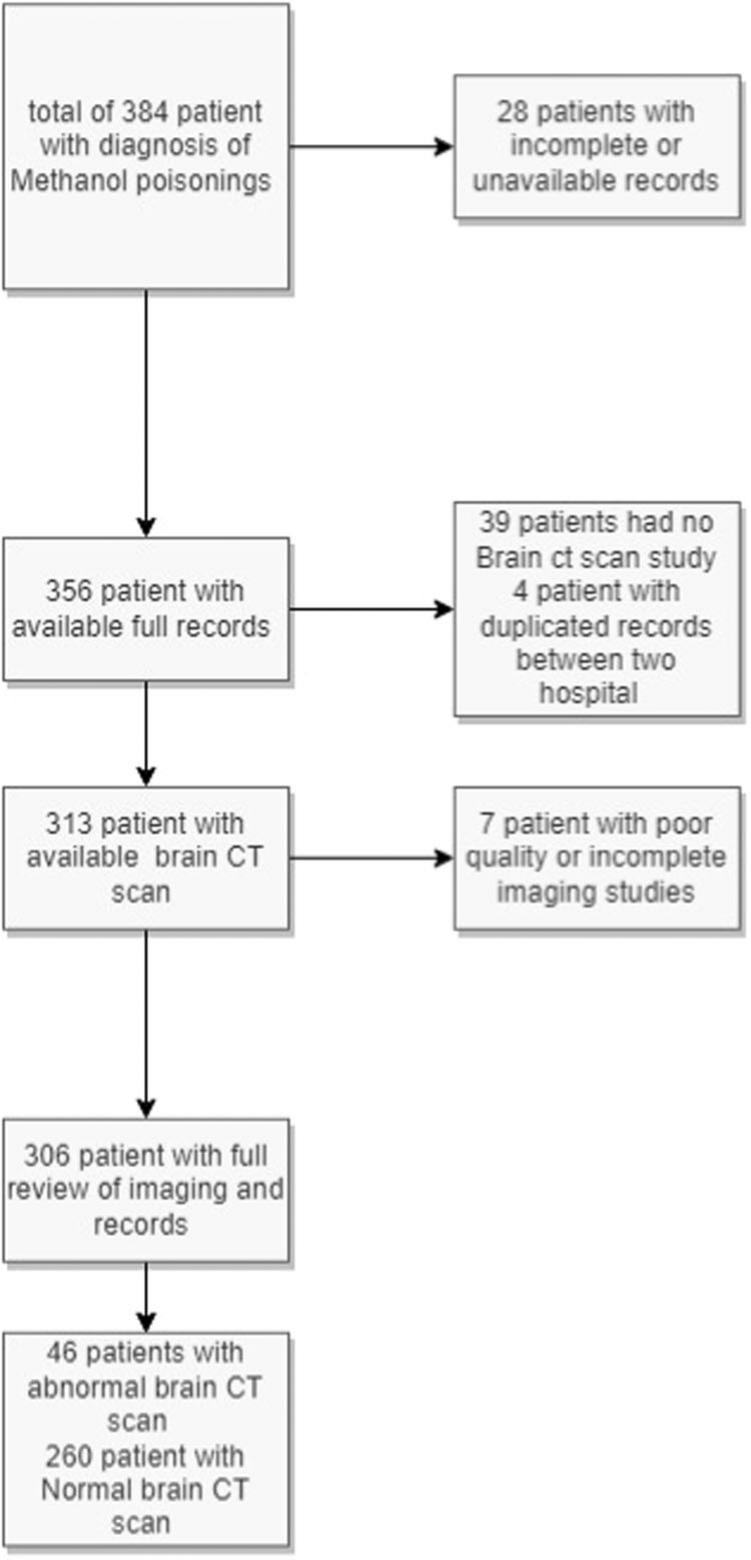
Flow chart of the study.

We conducted a retrospective cross‐sectional study of patients with confirmed methanol poisoning who were admitted to two tertiary‐care hospitals (Faghihi and Namazi General Teaching Hospitals) in Shiraz, Iran, during the COVID‐19 pandemic in March and April 2020 (Figure 1). The study was approved by the ethics committee of Shiraz University of Medical Sciences, and informed consent was obtained from the patients or their legal guardians. The diagnosis of methanol poisoning was based on a positive history of methanol ingestion and the presence of clinical signs of methanol toxicity, such as hyperventilation, altered mental status, visual disturbances, and high anion gap metabolic acidosis. We excluded patients who had no clinical evidence of methanol toxicity, chronic alcohol abuse or dependence, pre‐existing visual impairment or optic neuropathy from other causes, co‐ingestion of other toxic alcohols or substances, prior antidotal therapy, or hemodialysis, or incomplete or missing data on clinical, laboratory, or brain imaging characteristics or outcomes. We also excluded patients who had impaired medical images in the Picture Archiving and Communication System.

We collected data on demographic characteristics, medical history, clinical symptoms, vital signs, duration and amount of methanol consumption, Glasgow Coma Scale (GCS) score, outcome (mortality or survival), and sequelae (visual impairment or blindness) from hospital records. We measured arterial blood gas parameters (pH, bicarbonate [meq/L], and oxygen saturation [%]), creatinine level (mg/dL), blood glucose level (mg/dL), and electrolyte levels (sodium [meq/L], potassium [meq/L], magnesium [mg/dL], and calcium [mg/dL]) from blood samples. We assessed visual acuity using the Snellen chart, and defined blindness as a visual acuity below 20/20 in both eyes. The outcome was defined as mortality or survival at the time of discharge.

The brain CT scans of the patients from both centers were independently reviewed by three clinical radiologists using a pre‐designed checklist. The checklist included the presence or absence of hemorrhage, hypodensity in the basal ganglia, white matter, cortex, cerebellum, and extra‐axial regions, as well as the signs of diffuse cerebral edema, white matter edema, IVH, subdural hematoma, epidural hematoma, subarachnoid hemorrhage (SAH), and pseudo‐SAH. Additionally, the presence of hyper attenuation within the parenchyma or basal ganglia should be considered when evaluating for IVH, subdural hematoma, epidural hematoma, SAH, and intracerebral hemorrhage (ICH).^17^ Pseudo‐SAH is characterized by symmetrical high densities in the sulci and basal cistern, as shown in several slices.^18^ The radiologists were blinded to each other's assessments and to the clinical data of the patients. The interobserver agreement was calculated using Cohen's kappa coefficient. A consensus discussion was held to determine the final results of the CT scans. A subset of eight patients underwent brain magnetic resonance imaging (MRI) to further evaluate the brain lesions.

Data analysis was performed using SPSS software (version 22). Continuous variables were expressed as mean ± SD, and categorical variables were expressed as frequency and percentage. The normality of the data was assessed using the Kolmogorov–Smirnov test. Fisher's exact test was used to compare categorical variables, and Pearson's correlation test was used to examine the relationship between numerical variables. Correlation coefficients and *p* values were reported to quantify the strength and significance of the associations. A *p* value less than 0.05 was considered statistically significant.

## 3 RESULTS

A comprehensive examination was conducted on a cohort of 306 patients, all of whom were subsequently enrolled in the study (see Figure 1). During the enrollment period, a total of 268 individuals (88.88%) identified as male, while 34 individuals (11.11%) identified as female. The average age of the patients upon admission was 32.10 ± 9.9 years, ranging from 15 to 72 years. Out of the total, 179 individuals (58.5%) were admitted to Namazi Hospital, while 127 individuals (41.5%) were admitted to Faghihi Hospital. Upon admission, 65 patients, accounting for 21.8% of the sample, were found to be in an unconscious state as indicated by a GCS score below 8. The average GCS score for these patients was calculated to be 12.44 ± 4.5. The average length of hospitalization for patients was 2.72 ± 2.46 days, ranging from 1 to 14 days. Each patient underwent a brain CT scan, with a frequency ranging from 1 to 3 times (mean: 1.14 ± 0.44 times). Out of the overall sample size, 60 patients, accounting for 19.9% of the population, experienced mortality. The analysis of demographic data indicated that a total of 223 patients, accounting for 75.9% of the sample, exhibited symptoms of blindness. Additionally, it was found that 128 patients, representing 49.2% of the sample, had a history of chronic alcohol consumption. The prevalence of opium addiction was observed in 28 patients, accounting for 9.6% of the total sample. The laboratory data indicated pH (7.12 ± 0.21), HCO_3_ (10.98 ± 7.98 meq/L), O_2_ saturation (90.04 ± 10.96%), BUN (14.23 ± 11.50), Cr (1.44 ± 0.66 mg/dL), K (4.85 ± 1.21), Ca (9.53 ± 0.79), Mg (2.42 ± 1.64), and blood sugar (144.92 ± 98.40).

**Figure 2:**
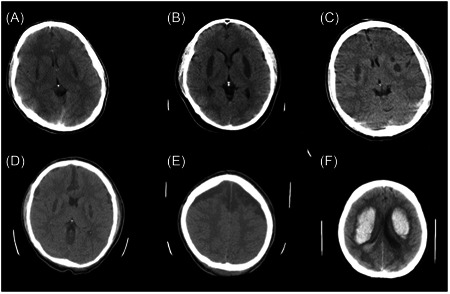
Brain CT scans can reveal the effects of methanol poisoning on the brain. The most common findings are hypodense lesions in the bilateral putamen, which may be symmetrical or asymmetrical (A–C). Few patients may also have subdural fluid collections, which are blood or cerebrospinal fluid accumulations between the dura mater and the arachnoid mater (D–E). These may compress the brain and cause neurological deficits or death. A rare but severe complication of methanol poisoning is hemorrhage in the putamen and the ventricles (F). This occurs from necrosis and rupture of the blood vessels in the affected areas. Patients with this condition usually have a low Glasgow Coma Scale score and seizures, and have a poor prognosis. CT, computed tomography.

The findings from the brain computed tomography (CT) scan imaging revealed that only 46 individuals, constituting 15% of the sample, displayed abnormal results. In contrast, within the population of patients who died, it was observed that 30 individuals, accounting for 50% of the sample, exhibited normal findings on their CT scans. Table 1 presents a complete depiction and comparative analysis of all CT scan observations. Regarding the findings observed in the brain CT scan, Out of the five observed cases of ICH, four individuals were diagnosed with putamen hemorrhage, and unfortunately, all of them experienced fatal outcomes. In contrast, only one instance displayed hemorrhage in the Globus pallidus and exhibited a positive prognosis. Additionally, there was a single patient who presented with IVH together with considerable bilateral putamen hemorrhage, as illustrated in Figure 2. All of the patients with pseudo‐SAH had concurrent scattered cerebral edema. A noteworthy finding outside of the parenchyma was the detection of subdural fluid collection, which was only observed in two cases. In one instance, a small quantity of fluid was seen on the right temporal side, suggesting a positive prognosis. In contrast, the second case demonstrated a remarkable buildup of subdural fluid, which displayed a gradual increase over a duration of 6 h as shown in two consecutive CT images. Table 2 analyses the correlation between demographic and laboratory variables and brain imaging outcomes. Notable correlations were noted between the two cohorts in various items including pH, GCS score, renal failure, bicarbonate (HCO_3_) levels, arterial partial pressure of oxygen (PaO_2_), blood glucose levels, potassium (K) levels, base excess, and arterial partial pressure of carbon dioxide (PaCO_2_) (*p* < 0.05).

**Table 1:** Comparison of imaging findings with the patient's survival status.

	Status		Total	*p* Value
	Live	Death		
Diffused cerebral edema	4 (1.6%)	19 (31.7%)	23 (7.5%)	<0.001
Globus palladium	2 (0.8%)	4 (6.7%)	6 (2%)	<0.001
Putamen	14 (5.7%)	20 (33.3%)	34 (11.11%)	<0.001
White matter Edema	1 (0.4%)	10 (16.7%)	11 (3.6%)	<0.001
Cerebellum nucleus hypodensity	0 (0%)	25 (41.7%)	25 (8.2%)	<0.001
ICH	1 (0.4%)	4 (6.7%)	5 (1.6%)	<0.001
Pseudo‐SAH	2 (0.8%)	8 (13.3%)	10 (3.3%)	<0.001

Abbreviations: ICH, intracerebral hemorrhage; SAH, subarachnoid hemorrhage.

**Table 2:** Comparison of demographic and laboratory data with brain imaging results.

Variable	Brain imaging		*p* Value
	Normal	Abnormal	
Age (years)	31.88 ± 10.0	33.3 ± 9.52	0.35
Sex (male/female)	234/26	38/8	0.14
Chronic alcohol user (yes/no)	112/109	16/23	0.67
Opium addiction (yes/no)	25/223	3/42	0.47
Renal failure (yes/no)	91/165	30/13	<0.001
GCS (score)	13.22 ± 3.9	7.95 ± 5.40	<0.001
PH (unit)	7.16 ± 0.20	6.93 ± 0.22	<0.001
PaO_2_ (mmHg)	91.56 ± 9.66	81.69 ± 13.76	<0.001
PaCO_2_ (mmHg)	13.01 ± 0.81	17.93 ± 2.64	0.02
HCO_3_ (mEq/L)	11.29 ± 7.24	9.2 ± 11.19	<0.001
Base excess (mEq/L)	−1.2 ± −1.8	−3.4 ± −2.6	<0.001
Na (mEq/L)	142.17 ± 4.03	142.90 ± 5.65	0.43
K (mEq/L)	4.74 ± 1.10	5.54 ± 1.55	<0.001
Blood sugar (mg/dL)	134.82 ± 74.40	200.34 ± 158.21	0.01

Table 3 presents a comparison between the primary demographic and laboratory data and the final patient status. The study findings indicate that those below the age of 30 had a significantly greater likelihood of surviving compared to those aged 60 years and above (odds ratio [OR] = 3.21, 95% confidence interval [CI] = 1.23–8.36, *p* = 0.017). Male patients exhibited a significantly reduced likelihood of survival compared to female patients (OR = 0.42, 95% CI = 0.19–0.93, *p* = 0.032). Those with blindness had a significantly reduced likelihood of survival compared to those without blindness (OR = 0.16, 95% CI = 0.07–0.38, *p* < 0.001). Individuals with opium addiction had a significantly reduced likelihood of survival compared to those without opium addiction (OR = 0.27, 95% CI = 0.12–0.61, *p* = 0.002). Individuals who were classified as chronic alcohol users had a significantly reduced likelihood of survival compared to those who did not have a history of chronic alcohol use (OR = 0.29, 95% CI = 0.13–0.66, *p* = 0.003). Those with elevated blood sugar levels had a significantly reduced likelihood of survival compared to those with normal blood sugar levels (OR = 0.31, 95% CI = 0.14–0.69, *p* = 0.004). The likelihood of survival was significantly lower among patients with abnormal CT scans compared to those with normal CT scans (OR = 0.18, 95% CI = 0.08–0.41, *p* < 0.001).

**Table 3:** Comparison of demographical data based on survival status and imaging data.

Variable		Status		Odds ratio (95% CI)	Odds ratio (95% CI)	*p* Value
		Alive	Death			
Age	Less than 30 years	132 (53.7%)	17 (28.8%)	1	1	–
	30–40 years	83 (33.7%)	23 (39%)	2.15 (1.09–4.27)	1.83 (0.44–7.64)	0.028
	More than 40 years	31 (12.6%)	19 (32.2%)	4.76 (2.22–10.20)	2.56 (0.51–12.73)	<0.001
Sex	Male	218 (88.6%)	54 (90.0%)	1.16 (0.46–2.93)	0.71 (0.10–4.84)	0.760
	Female	28 (11.4%)	6 (10.0%)	1	1	–
Blindness		172 (71.7%)	51 (94.4%)	6.72 (2.02–22.26)	1.74 (0.299–10.22)	0.002
Chronic alcohol user		108 (52.2%)	20 (37.7%)	0.55 (0.29–1.03)	2.40 (0.55–10.22)	0.063
Opium addiction		25 (10.7%)	3 (5.1%)	0.44 (0.13–1.53)	0.04 (0.00–2.02)	0.219
Renal failure		73 (30.2%)	48 (84.2%)	12.37 (7.02–29.41)	4.71 (1.22–18.10)	<0.001
O2 sat %		93.89 ± 5.37	74.14 ± 13.06	23.78 (6.63–89.07)	10.19 (2.31–44.91)	<0.001
Cr (mg/dL)		1.29 ± 0.41	2.11 ± 1.03	0.03 (0.01–0.07)	7.96 (1.35–46.95)	<0.001
Blood sugar		125.98 ± 74.40	222.87 ± 139.64	22.77 (9.63–53.87)	11.13 (1.79–68.90)	<0.001
Abnormal CT scan		16 (6.5%)	30 (50%)	14.37 (7.02–29.41)	15.55 (3.97–60.88)	<0.001

Abbreviations: CI, confidence interval; CT, computed tomography.

## 4 DISCUSSION

The purpose of this study was to investigate the main clinical and laboratory data manifestations that were associated with the survival of the patients. Most of the previous studies reported the clinical and paraclinical findings in methanol poisoning cases, such as blindness, severe anion‐gap metabolic acidosis, and gastrointestinal symptoms (such as abdominal pain, nausea, and vomiting).^5^, ^6^, ^8^, ^9^, ^10^, ^19^, ^20^ However, no previous study examined the relationship between symptoms and survival. Moreover, our study was the largest study on methanol poisoning and imaging findings. In our study, among multiple clinical signs and symptoms among our patients, we found that age greater than 30 years and a history of blindness (75.9%) were significantly associated with poor patient survival. However, neither of these factors had any relation with abnormal imaging findings. We also found no correlation between sex, gastrointestinal symptoms, seizures, and patient prognosis and brain imaging findings. Among the laboratory data, a GCS score less than 8, pH less than 7.2, oxygen saturation (O_2_ sat) less than 90%, creatinine (Cr) greater than 1.9 mg/dL, potassium (K) greater than 7 mg/dL, bicarbonate level, and hyperglycemia were significantly associated with poor prognosis. However, only a GCS score of less than 8, pH less than 7.2, O_2_ sat less than 90%, and hyperglycemia were associated with abnormal imaging findings.

In addition, we discovered that opium addiction or chronic alcohol use had no correlation or protective effect on methanol poisoning. This contradicts a widespread misconception among some patients and doctors.^21^ Other studies have reported acute kidney injury as a very specific presentation of methanol poisoning, a finding that we have corroborated in this study.^22^

Putamen hypodensity was the most frequent imaging finding, observed in 11.11% of cases. This was followed by cerebellum nucleus hypodensity (8.2%) and diffused white matter edema (7.5%). Notably, there was a significant correlation between cerebellum nucleus hypodensity and diffused cerebral edema, with about 68% of cases presenting both findings. Our data align with the study by Simani et al. in Tehran, as well as a recent systematic review that reported similar results.^12^, ^19^ In terms of prognosis, cerebellum nucleus hypodensity, intracranial hemorrhage (ICH), and pseudo‐SAH were associated with poor outcomes. Almost all previous studies have reported that intracranial events, especially ICH, are associated with poor prognosis. For instance, in the study by Simani et al., all eight patients with ICH died. They concluded that putaminal or subcortical white matter hemorrhage, as well as lower initial GCS and bicarbonate levels, are associated with higher mortality rates.^12^ Simani et al. reported unilateral and bilateral putaminal necrosis in 5% and 40% of their patients, respectively. Another prospective study by Taheri et al. showed that bilateral putaminal hypodense lesions (96.4%) were the most prevalent brain CT scan findings, followed by putaminal hemorrhage (25%) and hypo‐attenuated white matter lesions (21.4%). These findings confirm the results of our study.^16^ They also reported that both putaminal hemorrhage and subcortical necrosis of the insula were significantly associated with death in these patients. In our study, we encountered two cases of subdural fluid collection, a finding that has not been previously reported. This rare finding could support the theory of hypotension and decreased blood supply in methanol poisonings.^23^, ^24^ There are multiple theories about the effect of putaminal involvement in methanol toxicity. Some literature suggests that high concentrations of formic acid led to decreased blood flow within basal veins, causing ischemic changes or hemorrhagic necrosis in the deep part of the brain, including the basal ganglion or cerebellum nucleus.^6^, ^14^, ^25^

We found two cases of subdural fluid collection, which was not reported before. This rare finding may show that low blood pressure and blood supply affect methanol poisonings.^23^, ^24^ There are different theories about how putaminal involvement causes methanol toxicity. Some studies say that formic acid reduces blood flow in basal veins, leading to ischemia or bleeding in deep brain areas like basal ganglia or cerebellum nucleus.^6^, ^14^, ^25^

Sefidbakht et al. suggested a triad of visual impairment, gastrointestinal symptoms, and metabolic acidosis occurring within 6–24 h.^5^ However, based on our experience and the results of the present study, we propose that any patient with high anion gap acidosis, a GCS score less than 8, pH less than 7.2, oxygen saturation (O_2_ sat) less than 90%, and hyperglycemia should undergo a brain CT scan as a first priority. Other patients could undergo a CT scan at an appropriate time, especially during outbreaks.

In our study, the most common finding was a normal CT scan, observed in 85% of patients. This suggests that the criteria for ordering a brain CT scan in cases of methanol poisoning are not clearly defined, indicating the need for a well‐designed system. Interestingly, approximately 50% of the patients who passed away had normal CT scan findings. Furthermore, only 46 patients (15%) presented with abnormal brain CT scans, and about 34.78% (16 patients) of them survived. A similar study conducted by Simani et al., concurrently in Tehran, reported that out of 516 patients, only 40 underwent a brain CT scan. Among these, only eight patients (20%) had normal brain CT scans, all of whom survived.^26^ Another previous study in Iran showed that 66.6% of patients who underwent a CT scan due to acute methanol poisoning had abnormal findings in their brain CT scans.^16^ Most other studies did not have sample sizes as large as ours and were mostly case reports and case series studies.^3^, ^4^, ^13^, ^15^ One possible explanation for the high number of normal CT scans in our study is the concurrent outbreaks of methanol and COVID‐19 during our study period, which resulted in ordering a CT scan as a routine procedure in the first line of examination in emergency departments by interns and residents. This led to performing CT scans at a very early stage of admission, before the brain damage became apparent. Moreover, due to overcrowding in the imaging ward, each patient could only have one CT scan in the early hours or days of admission and could not have a delayed or follow‐up CT scan. Another possible explanation for the normal CT scans in our study is that the cause of death among the deceased patients could be damage to other organ systems, such as cardiac,^27^ renal failure, and other organs,^19^ rather than brain injury. Therefore, imaging may not be very useful at the early stage of admission. Instead, imaging should be planned to estimate prognosis in combination with laboratory data. our experience in this study demonstrated that during such methanol poisoning outbreaks, a CT scan was requested for most patients with any history of suspected methanol poisoning. This led to overcrowding in the emergency and imaging wards. We also suggest designing a scoring system that combines imaging findings and lab data to decide on the next therapeutic plan for patients.

We evaluated the correlation between significant findings from brain CT scans and symptoms. Our analysis revealed a significant association between ICH and sudden blindness. However, no association was found between ICH and other symptoms. We also discovered that putamen hypodensity elevates the risk of entering a coma state and experiencing seizures, but it does not correlate with the occurrence of other symptoms. The incidence of coma state, seizures, and vomiting was higher (though not statistically significant) among patients exhibiting diffused cerebral edema in their CT scan imaging.

MRI images of our patients did not reveal more findings than CT scans. Sefidbakht et al., in a letter to the editor, suggested that CT scans and MRIs can reveal changes in patients with methanol poisoning, which could be helpful for physicians. However, we do not recommend MRI during the acute phase of methanol poisoning.^5^ Post‐ischemic changes, such as small cystic changes, may be present in the brain. Follow‐up MRI or magnetic resonance spectroscopy may be beneficial for evaluating patients with permanent or temporary neurological symptoms. Further studies should be established to follow up on methanol toxicity.^3^, ^28^ Although our experience with some methanol cases showed mostly normal findings on follow‐up CT scans, we recommend follow‐up MRI only in cases with high‐degree abnormal findings on the initial CT scan.

Despite advancements in the diagnosis and treatment of methanol toxicity, the final morbidity and mortality rates remain high.^7^, ^9^, ^10^, ^26^ In our study, we found a mortality rate of approximately 19.9% due to methanol toxicity. Recent studies have reported varying mortality rates ranging from 0% to 48%, which aligns with our findings.^10^, ^12^, ^25^, ^29^ In the study by Chang et al., the majority of patients were male and habitual alcohol consumers, similar to our study. However, their patients were, on average, about 15 years older than ours. They reported blindness and photophobia as the least common symptoms, occurring in 10% and 4% of patients, respectively. The most common symptoms were respiratory symptoms (60%) and nausea and vomiting (42%). These results are consistent with our study. Blurred vision was reported more frequently by our patients (32%) than those in Chang et al.'s study. As Asians, Taiwanese have cultural similarities with Iranians compared to people in the United States.^29^ In contrast to our study, Kaewput et al. found that 44% of their patients with a similar mean age had used methanol for suicide. Visual impairment and optic neuritis were among the most prevalent symptoms in their patients (8%). The mean hospital stay in Kaewput et al.'s study was4 days, slightly longer than ours, which was 3 days.^25^

Our study had some limitations. First, due to its retrospective design and the inadequacy of the patient record system, we faced data and variable scarcity. We could not obtain data on respiratory symptoms, the time interval from methanol ingestion to emergency department admission, and history of alcoholic beverage consumption, which could have influenced the clinical course and outcome of methanol poisoning. Second, we did not perform any follow‐up for the patients after discharge, so we could not assess the long‐term effects of methanol poisoning on their health and quality of life. Third, we did not measure the serum methanol level or the osmolal gap in our patients, which are important diagnostic and prognostic markers of methanol poisoning. Fourth, we did not include a control group of patients without methanol poisoning to compare the brain imaging findings and to rule out other causes of brain lesions.

In conclusion, this study showed that approximately 15% of patients with methanol poisoning had abnormal brain CT scan findings, with putamen hypodensity, cerebellar nucleus hypodensity, and diffuse cerebral edema being the most common. These findings were significantly associated with poor prognosis, as well as ICH. Blindness was the most prognostic clinical sign, while renal failure, GCS score less than 8, pH less than 7.2, and hyperglycemia were the most reliable laboratory data for both prognosis and abnormal brain findings. This suggests that a normal brain CT scan is not a reliable indicator of the severity or prognosis of methanol poisoning. Therefore, clinicians should use clinical examination and laboratory tests to evaluate methanol‐poisoned patients.

## AUTHOR CONTRIBUTIONS

**Saeid Esmaeilian**: Conceptualization; data curation; formal analysis; investigation; methodology; project administration; software; supervision; visualization; writing—original draft; writing—review and editing. **Arash Teimouri**: Data curation; formal analysis; methodology; project administration; software; writing—original draft; writing—review and editing. **Sedighe Hooshmandi**: Data curation; investigation; methodology; project administration; supervision; writing—original draft; writing—review and editing. **Mohammad Hossein Nikoo**: Data curation; methodology; project administration; supervision; writing—original draft; writing—review and editing. **Seyed Taghi Heydari**: Conceptualization; methodology; project administration; software; supervision; validation; writing—original draft; writing—review and editing. **Elham Mohajeri**: Conceptualization; data curation; methodology; project administration; software; validation; writing—original draft; writing—review and editing. **Sina Bazmi**: Data curation; formal analysis; investigation; methodology; project administration; visualization; writing—original draft; writing—review and editing. **Reza Tabrizi**: Investigation; methodology; project administration; supervision; validation; writing—original draft; writing—review and editing. **Meisam Hoseinyazdi**: Conceptualization; investigation; methodology; resources; supervision; visualization; writing—original draft; writing—review and editing.

## CONFLICT OF INTEREST STATEMENT

The authors declare no conflict of interest.

## ETHICS STATEMENT

Ethical approval to report this case was obtained from the Ethics Committee of Shiraz University of Sciences (approval no. IR.SUMS.REC.1399.387).

## TRANSPARENCY STATEMENT

The lead author Seyed Taghi Heydari affirms that this manuscript is an honest, accurate, and transparent account of the study being reported; that no important aspects of the study have been omitted; and that any discrepancies from the study as planned (and, if relevant, registered) have been explained.

## Data Availability

The data supporting this manuscript's findings are not publicly available due to Ethical restrictions. Data are, however, available from the authors upon reasonable request and with permission of Shiraz University of Sciences.

## References

[1] Liberski S , Kaluzny BJ , Kocięcki J . Methanol‐induced optic neuropathy: a still‐present problem. Arch Toxicol. 2022;96:431‐451. 34988610 10.1007/s00204-021-03202-0 PMC8731680

[2] Gallagher N , Edwards FJ . The diagnosis and management of toxic alcohol poisoning in the emergency department: a review article. Adv J Emerg Med. 2019;3(3):28. 10.22114/ajem.v0i0.153 PMC6683589 31410405

[3] Boukobza M , Baud FJ . Hemorrhagic infarct of basal ganglia in cardiac arrest. CT and MRI findings. 2 cases. Neurol Neurochir Pol. 2018;52(1):94‐97. 28965668 10.1016/j.pjnns.2017.09.004

[4] Khan BM , Alamgir W , Fatima T . Role of neuroimaging in methanol toxicity—a case report. Pak Armed Forces Med J. 2019;69(2):429.

[5] Sefidbakht S , Lotfi M , Jalli R , Moghadami M , Sabetian G , Iranpour P . Methanol toxicity outbreak: when fear of COVID‐19 goes viral. Emerg Med J. 2020;37(7):416. 32414710 10.1136/emermed-2020-209886 PMC7413583

[6] Iranpour P , Firoozi H , Haseli S . Methanol poisoning emerging as the result of COVID‐19 outbreak; radiologic perspective. Academic Radiol. 2020;27(5):755‐756. 10.1016/j.acra.2020.03.029 PMC7136884 32273134

[7] Hassanian‐Moghaddam H , Zamani N . A brief review on toxic alcohols: management strategies. Iran J Kidney Dis. 2016;10(6):344‐350. 27903992

[8] Hassanian‐Moghaddam H , Nikfarjam A , Mirafzal A , et al. Methanol mass poisoning in Iran: role of case finding in outbreak management. J Public Health. 2015;37(2):354‐359. 10.1093/pubmed/fdu038 24944254

[9] Aghababaeian H , Araghi Ahvazi L , Ostadtaghizadeh A . The methanol poisoning outbreaks in Iran 2018. Alcohol Alcohol. 2019;54(2):128‐130. 30715164 10.1093/alcalc/agz005

[10] Massoumi G , Saberi K , Eizadi‐Mood N , Shamsi M , Alavi M , Morteza A . Methanol poisoning in Iran, from 2000 to 2009. Drug Chem Toxicol. 2012;35(3):330‐333. 22289573 10.3109/01480545.2011.619193

[11] Choi J‐H , Lee SK , Gil Y‐E , et al. Neurological complications resulting from non‐oral occupational methanol poisoning. J Korean Med Sci. 2017;32(2):371‐376. 28049252 10.3346/jkms.2017.32.2.371 PMC5220007

[12] Simani L , Ramezani M , Roozbeh M , Shadnia S , Pakdaman H . The outbreak of methanol intoxication during COVID‐19 pandemic: prevalence of brain lesions and its predisposing factors. Drug Chem Toxicol. 2022;45:1500‐1503. 33172326 10.1080/01480545.2020.1845192

[13] Blanco M , Casado R , Vázquez F , Pumar JM . CT and MR imaging findings in methanol intoxication. Am J Neuroradiol. 2006;27(2):452‐454. 16484428 PMC8148792

[14] Camurcuoglu E , Halefoglu AM . CT and MR imaging findings in methanol intoxication manifesting with BI lateral severe basal ganglia and cerebral involvement. J Belg Soc Radiol. 2022;106(1):66. 35859917 10.5334/jbsr.2836 PMC9267022

[15] Naito H , Kurashige T , Kobayashi M , Naito K , Naka H , Tokinobu H . Magnetic resonance spectroscopy after methanol poisoning. Neurol Clin Neurosci. 2016;4(3):112‐114.

[16] Taheri MS , Moghaddam HH , Moharamzad Y , Dadgari S , Nahvi V . The value of brain CT findings in acute methanol toxicity. Eur J Radiol. 2010;73(2):211‐214. 19101105 10.1016/j.ejrad.2008.11.006

[17] de Oliveira AM , Paulino MV , Vieira APF , et al. Imaging patterns of toxic and metabolic brain disorders. Radiographics. 2019;39(6):1672‐1695. 31589567 10.1148/rg.2019190016

[18] Shirota G , Gonoi W , Ikemura M , et al. The pseudo‐SAH sign: an imaging pitfall in postmortem computed tomography. Int J Legal Med. 2017;131(6):1647‐1653. 28730501 10.1007/s00414-017-1651-1

[19] Mousavi‐Roknabadi RS , Arzhangzadeh M , Safaei‐Firouzabadi H , et al. Methanol poisoning during COVID‐19 pandemic; a systematic scoping review. Am J Emerg Med. 2022;52:69‐84. 34883289 10.1016/j.ajem.2021.11.026 PMC8611855

[20] Rostrup M , Edwards JK , Abukalish M , et al. The methanol poisoning outbreaks in Libya 2013 and Kenya 2014. PLoS One. 2016;11(3):e0152676. 27030969 10.1371/journal.pone.0152676 PMC4816302

[21] Shadnia S , Rahimi M , Soltaninejad K , Nilli A . Role of clinical and paraclinical manifestations of methanol poisoning in outcome prediction. J Res Med Sci. 2013;18(10):865‐869. 24497857 PMC3897070

[22] Banagozar Mohammadi A , Delirrad M . Problems with methanol poisoning outbreaks in Iran. Alcohol Alcohol. 2019;54:338. 30957140 10.1093/alcalc/agz028

[23] Osborne KA , Shigeno T , Balarsky AM , et al. Quantitative assessment of early brain damage in a rat model of focal cerebral ischaemia. J Neurol Neurosurg Psychiatry. 1987;50(4):402‐410. 3585350 10.1136/jnnp.50.4.402 PMC1031873

[24] Schievink WI , Maya MM , Moser FG , Tourje J . Spectrum of subdural fluid collections in spontaneous intracranial hypotension. J Neurosurg. 2005;103(4):608‐613. 16266041 10.3171/jns.2005.103.4.0608

[25] Kaewput W , Thongprayoon C , Petnak T , et al. Inpatient burden and mortality of methanol intoxication in the United States. Am J Med Sci. 2021;361(1):69‐74. 32958166 10.1016/j.amjms.2020.08.014

[26] Simani L , Ramezani M , Roozbeh M , Shadnia S , Pakdaman H . The outbreak of methanol intoxication during COVID‐19 pandemic: prevalence of brain lesions and its predisposing factors. Drug Chem Toxicol. 2022;45(4):1500‐1503. 33172326 10.1080/01480545.2020.1845192

[27] Nikoo MH , Estedal A , Pakfetrat M , Abtahi F , Heydari ST . Useful Electrocardiographic and Laboratory Findings in Shiraz Outbreak of 356 Methanol Poisoned Patients. 2020.

[28] Kufner A , Khalil AA , Galinovic I , et al. Magnetic resonance imaging‐based changes in vascular morphology and cerebral perfusion in subacute ischemic stroke. J Cereb Blood Flow Metab. 2021;41(10):2617‐2627. 33866849 10.1177/0271678X211010071 PMC8504415

[29] Chang S‐T , Wang Y‐T , Hou Y‐C , et al. Acute kidney injury and the risk of mortality in patients with methanol intoxication. BMC Nephrol. 2019;20(1):205. 31170938 10.1186/s12882-019-1404-0 PMC6554873

